# Exchange factors directly activated by cAMP mediate melanocortin 4 receptor-induced gene expression

**DOI:** 10.1038/srep32776

**Published:** 2016-09-09

**Authors:** Evi Glas, Harald Mückter, Thomas Gudermann, Andreas Breit

**Affiliations:** 1 Walther-Straub-Institut für Pharmakologie und Toxikologie, Ludwig-Maximilians-Universität., München, 80336 München, Germany.

## Abstract

G_s_ protein-coupled receptors regulate many vital body functions by activation of cAMP response elements (CRE) via cAMP-dependent kinase A (PKA)-mediated phosphorylation of the CRE binding protein (CREB). Melanocortin 4 receptors (MC4R) are prototypical G_s_-coupled receptors that orchestrate the hypothalamic control of food-intake and metabolism. Remarkably, the significance of PKA for MC4R-induced CRE-dependent transcription in hypothalamic cells has not been rigorously interrogated yet. In two hypothalamic cell lines, we observed that blocking PKA activity had only weak or no effects on reporter gene expression. In contrast, inhibitors of exchange factors directly activated by cAMP-1/2 (EPAC-1/2) mitigated MC4R-induced CRE reporter activation and mRNA induction of the CREB-dependent genes c-fos and thyrotropin-releasing hormone. Furthermore, we provide first evidence that extracellular-regulated kinases-1/2 (ERK-1/2) activated by EPACs and not PKA are the elusive CREB kinases responsible for MC4R-induced CREB/CRE activation in hypothalamic cells. Overall, these data emphasize the pivotal role of EPACs rather than PKA in hypothalamic gene expression elicited by a prototypical G_s_-coupled receptor.

MC4R are activated by melanocortins such as the α-melanocyte-stimulating hormone (α-MSH). They are predominantly expressed in the brain, but also in adipocytes, melanocytes as well as in the heart, lung, liver and testis^1^,^2^,^3^,^4^,^5^,^6^. MC4R signalling induces the expression of a set of specific genes to exert catabolic effects by decreasing food intake and increasing energy expenditure. In addition, MC4R exert anti-inflammatory actions by means of decreased cytokine expression and prostaglandin release^7^. Furthermore, α-MSH-induced MC4R activation has been shown to be neuroprotective, to improve memory and learning, induce neurite-like outgrowth, and affect reproduction^8^,^9^,^10^,^11^,^12^,^13^.

MC4R belong to the superfamily of G protein-coupled receptors (GPCR). In analogy to β-adrenergic receptors they regulate intracellular cAMP concentrations by G_s_ protein-mediated adenylyl cyclase activation^14^. As expected from a prototypical G_s_-coupled receptor, MC4R have been shown to modify the activity of multiple kinases such as PKA, AMP-activated kinase, c-jun kinase, phosphatidylinositol-3-kinase and protein kinase C^15^. Down-stream of these kinases, MC4R signalling regulates ion channel activity and gene expression^15^. Effects of MC4R on gene expression have so far been attributed to cAMP-mediated PKA activation leading to subsequent phosphorylation of the transcription factor CREB and CRE-dependent transcription^16^,^17^,^18^,^19^,^20^,^21^,^22^,^23^,^24^,^25^. However, it appears that the role of PKA in this process is conjectural rather than experimentally documented, because to date a definitive role of PKA for MC4R-induced CREB/CRE activation has not been rigorously worked out. This scenario is particularly remarkable because 1) ERK-1/2 kinases have been implicated in MC4R-induced hypothalamic CREB phosphorylation *in vivo* and 2) exchange factors directly activated by cAMP (so called EPAC proteins) induce CREB phosphorylation via ERK-1/2 when dopamine or cell-permeable cAMP analogues were applied to PC-12 or pituitary cells^26^,^27^,^28^,^29^,^30^,^31^. Thus, EPACs may represent an alternative molecular connection between MC4R/G_s_ and CREB/CRE. However, the role of EPACs for MC4R signaling has not yet been experimentally interrogated at all.

In order to dissect the roles of PKA and EPACs in MC4R-promoted CREB/CRE-dependent gene expression, we took advantage of HEK-293 cells and two distinct hypothalamic cell lines that either express recombinant human or endogenous murine MC4R and investigated the impact of selective pharmacological PKA, EPAC-1/2 or ERK-1/2 inhibition on MC4R-induced CREB phosphorylation, CRE activation and c-fos or TRH mRNA induction.

## Results

### Pivotal role of EPACs for α-MSH-induced CRE activation

**Figure 1:**
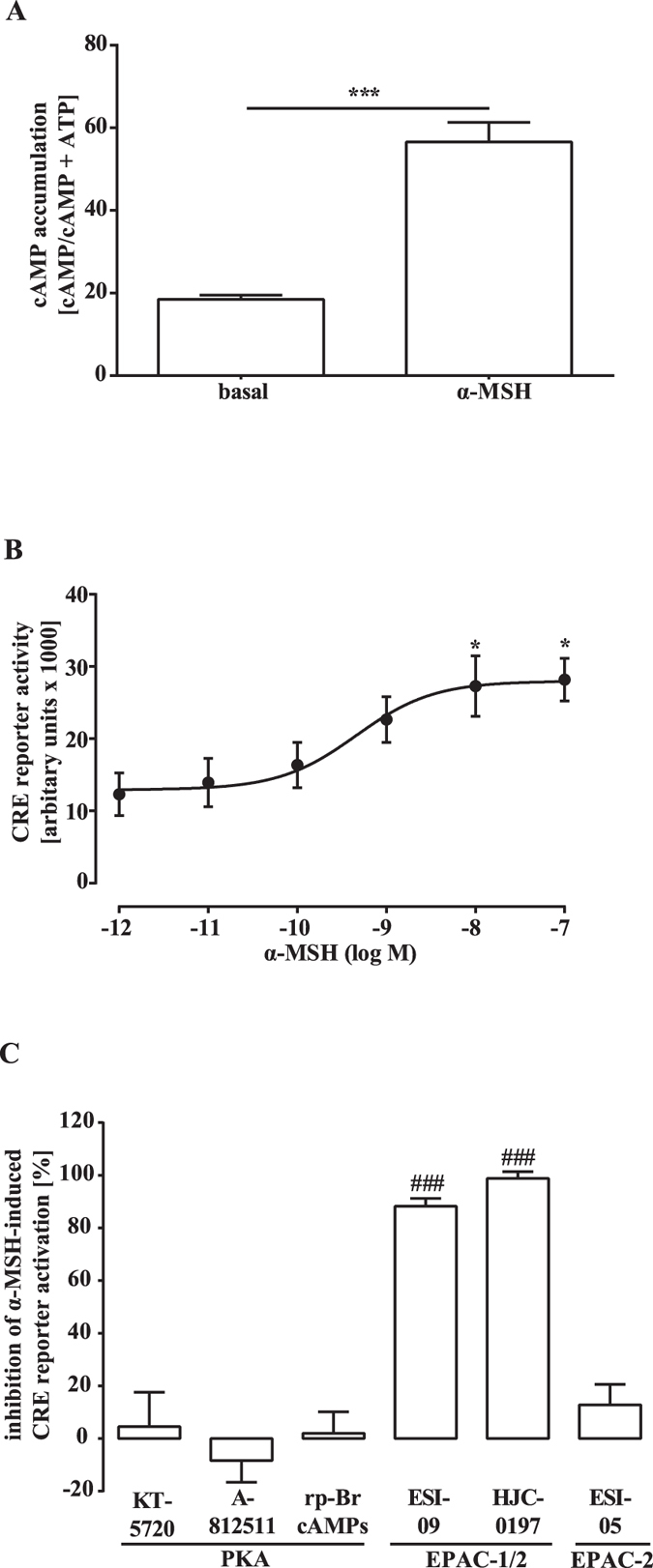
Significant role for EPACs in α-MSH-induced CRE activation: HEK-293-MC4R cells. (**A**) cAMP accumulation was measured after labeling of HEK-293-MC4R cells with [^3^H]-adenine followed by the purification of [^3^H]cAMP and [^3^H]ATP by sequential chromatography. Cells were stimulated with 1 μM α-MSH for 30 min at 37 °C (N = 5). Asterisks indicate a significant difference between MSH and basal using the two-sample Student’s t-test. In (**B**,**C**) HEK-293-MC4R cells were transfected with a reporter gene construct harboring the firefly luciferase gene under the control of a CRE-dependent promoter. In (**B**) cells were stimulated with various concentrations of α-MSH for 4 h. Means of 4 independent experiments performed in quadruplicates were compiled. Asterisks indicate a significant difference to cells stimulated with the lowest α-MSH concentration (−12) using one-way ANOVA followed by Dunnett’s analysis. In (**C**) cells were stimulated or not with 1 μM of α-MSH for 4 h after 30 min pretreatment with KT-5720 (5 μM; N = 7), A-812511 (10 μM; N = 8), rp-Br-cAMPs (50 μM; N = 6), ESI-09 (20 μM; N = 14), HJC-0197 (25 μM; N = 3) or ESI-05 (50 μM; N = 4) or the carrier DMSO (0.1% or 0.2%). Data were compiled first by normalizing α-MSH-induced CRE activation to the carrier or to the inhibitor alone and then by calculating the inhibition as percentage. Hashed signs indicate a significant difference to zero using the one-sample Student’s t-test.

To investigate the role of PKA and EPACs in MC4R-induced CRE activation, we used previously established HEK-293-MC4R cells stably expressing the human MC4R^32^. HEK-293-MC4R cells reacted to α-MSH with increased cAMP accumulation (Fig. 1A) and concentration-dependent CRE activation after transfection of cells with a CRE-dependent reporter plasmid (Fig. 1B). Thus, we used HEK-293-MC4R cells to analyse the effects of the PKA inhibitors KT-5720, A-812511 and rp-Br-cAMPs, the EPAC-1/2 inhibitors ESI-09 and HJC-0197 or the EPAC-2 selective inhibitor ESI-05 on MC4R-induced CRE activation (Fig. 1C and Suppl. S1). Surprisingly, none of the PKA inhibitors attenuated the effects of α-MSH on the CRE reporter, indicating that PKA activity is not required in this process. In contrast, both EPAC-1/2 inhibitors blunted α-MSH-induced CRE activation, compatible with the notion that EPAC-1/2 activity is required. The EPAC-2 selective inhibitor was without effect, suggesting that EPAC-1 is responsible for MC4R-mediated CRE activation in HEK-293 cells.

**Figure 2:**
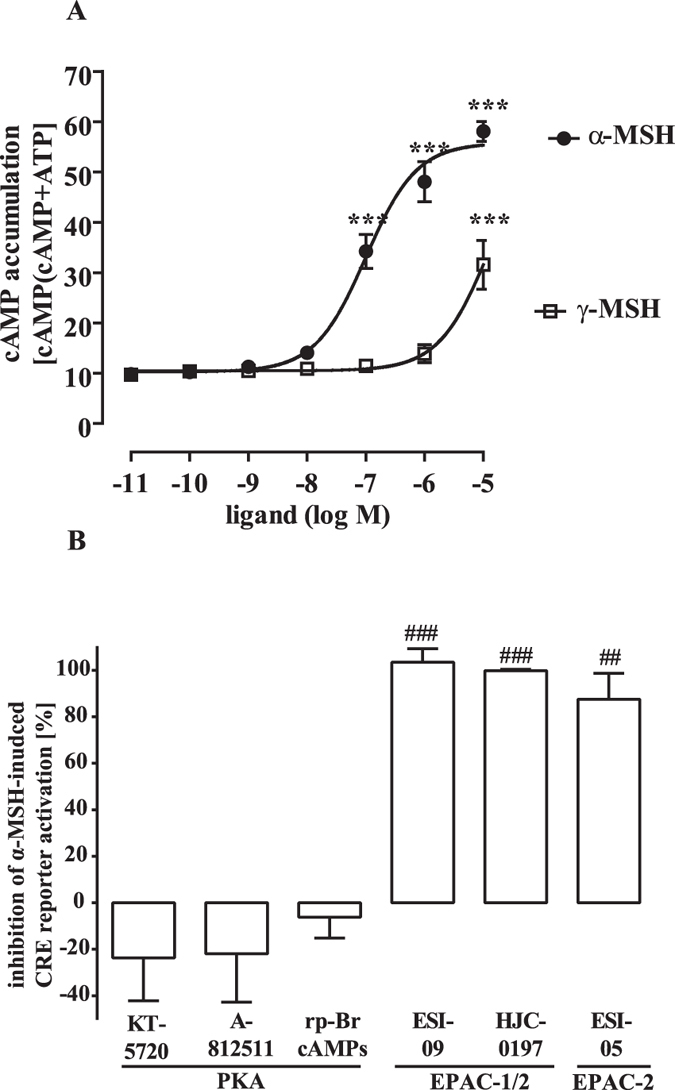
Significant role for EPACs in α-MSH-induced CRE activation: GT1-7 cells. (**A**) cAMP accumulation was measured after labeling of GT1-7 cells with [^3^H]-adenine followed by the purification of [^3^H]cAMP and [^3^H]ATP by sequential chromatography. Cells were stimulated with various concentrations of α-MSH and γ-MSH for 30 min at 37 °C. Means of 5 independent experiments performed in quadruplicates were compiled. Asterisks indicate a significant difference to cells stimulated either with the lowest concentration of α-MSH (−11) or γ-MSH (−9) using one-way ANOVA followed by Dunnett’s analysis. (**B**) GT1-7 cells were transiently transfected with a reporter gene construct harbouring the firefly luciferase gene under control of a CRE-dependent promoter. Cells were stimulated or not with 1 μM of α-MSH for 4 h after 30 min pretreatment with KT-5720 (5 μM; N = 7), A-812511 (10 μM; N = 4), rp-Br-cAMPs (50 μM; N = 5), ESI-09 (20 μM; N = 5), HJC-0197 (25 μM; N = 3) or ESI-05 (50 μM; N = 5) or the carrier DMSO (0.1% or 0.2%). Data were compiled first by normalizing α-MSH-induced CRE activation to the carrier or to the inhibitor alone and then by calculating the inhibition as percentage. Hashed signs indicate a significant difference to zero using the one-sample Student’s t-test.

We next asked whether the participation of EPACs in MC4R-induced CRE-dependent reporter activation is restricted to HEK-293 cells or rather reflects a general scenario. Therefore, we used the well-established, hypothalamic cell line GT1-7 endogenously expressing murine MC4R^33^. As shown in Fig. 2A, the GT1-7 cell pool used herein reacted to α-MSH with concentration-dependent cAMP accumulation, indicating endogenous expression of the MC4R or the MC3R subtype, which is also expressed in hypothalamic cells and activated by α-MSH. To distinguish between both MCR subtypes, we used γ-MSH activating MC3R, but not MC4R with a potency similar to α-MSH. The concentration-response curve of γ-MSH was shifted to higher concentrations by a factor of ~100, strongly suggesting expression of the MC4R in GT1-7 cells. Next, we transfected the CRE reporter into GT1-7 cells and analysed the effects of PKA and EPAC-1/2 inhibitors on α-MSH-induced CRE activation (Fig. 2B, and Suppl. S2). Concordant with the data observed in HEK-293-MC4R cells, none of the three PKA inhibitors affected CRE reporter activation, whereas both EPAC-1/2 inhibitors completely blocked MC4R-promoted gene transcription. Thus the involvement of EPACs in α-MSH-induced CRE activation is not a HEK-293 cell specific phenomenon, but rather a common feature of the MC4R. In contrast to HEK-293 cells, the EPAC-2 specific inhibitor ESI-05 also blocked α-MSH-induced CRE activation in GT1-7 cells underscoring cell-type specific roles of EPAC-1 and -2 in this process.

**Figure 3:**
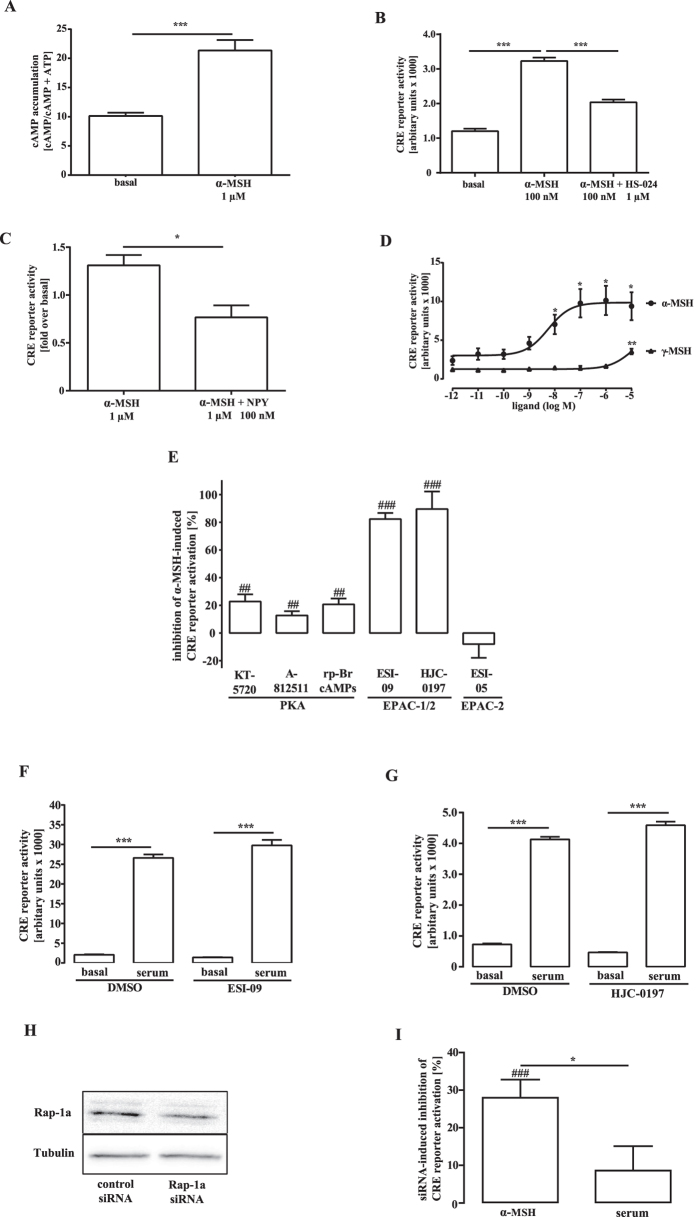
Significant role for EPACs in α-MSH-induced CRE activation: mHypoA-2/10-CRE cells. (**A**) Cells were stimulated with α-MSH for 30 min (N = 5). Asterisks indicate a significant difference using the two-sample Student’s t-test. (**B**) Cells were stimulated wit α-MSH or co-stimulated with HS-024 for 4 hours (N = 3). Asterisks indicate a significant difference using one-way ANOVA followed by Dunnett’s analysis. (**C**) Cells were pretreated or not with neuropeptide Y (NPY) and then stimulated with α-MSH. Data were compiled by calculating the x-fold over basal (N = 5). Asterisk indicates a significant difference using the two-sample Student’s t-test. (**D**) Cells were stimulated with increasing concentration of α-MSH or γ-MSH. Means of 11 (α-MSH) or 3 (γ-MSH) experiments performed in triplicates were compiled. Asterisks indicate a significant difference to cells stimulated with the lowest concentration using one-way ANOVA followed by Dunnett’s analysis. (**E**) Cells were stimulated or not with 1 μM of α-MSH for 4 h after 30 min pretreatment with KT-5720 (5 μM; N = 6), A-812511 (10 μM; N = 11), rp-Br-cAMPs (50 μM; N = 5), ESI-09 (20 μM; N = 14), HJC-0197 (25 μM; N = 5) or ESI-05 (50 μM; N = 10) or the carrier DMSO. Data were compiled by normalizing α-MSH-induced CRE activation to the carrier or to the inhibitor alone and then by calculating the inhibition as percentage. Hashed signs indicate a significant difference using the one-sample Student’s t-test. (**F**) Cells were preincubated with 0.2% DMSO or 20 μM ESI-09 and in (**G**) with 0.1% DMSO or 25 μM HJC-0197 for 30 min and then stimulated with 20% serum for 4 hours. Asterisks indicate a significant difference using two-way ANOVA followed by Bonferroni analysis. (**H**) Cells transfected with a specific Rap-1a or a control siRNA were subjected to western-blot analysis. In (**I**) α-MSH- (1 μM) or serum-induced (20%) CRE activation was determined in cells either transfected with 50 nM of a specific Rap-1a or a control siRNA. Data were compiled first normalized to control cells or cells transfected with the Rap-1a siRNA and then by calculating the inhibition as percentage (N = 5). Hashed signs indicate a significant difference using the one-sample Student’s t-test, asterisks using the two-sample Student’s t-test.

After having established a salient role of EPACs in MC4R-induced CRE-dependent activation, we next aimed at dissecting pertinent signaling pathways. In order to analyse CRE activity under similar experimental conditions over a longer time period, we took advantage of previously reported hypothalamic mHypoA-2/10 cells that endogenously express MC4R and have stably integrated the CRE reporter plasmid^24^. mHypoA-2/10-CRE cells reacted to α-MSH with elevated cAMP concentrations (Fig. 3A) and CRE activation inhibitable by the selective MC4R antagonist, HS-024 (Fig. 3B) or the physiological antagonist, neuropeptide Y (Fig. 3C), suggestive of the expression of functional MC4R in mHypoA-2/10-CRE cells. In line with this notion, the potency of γ-MSH was profoundly reduced when compared to α-MSH (Fig. 3D). Therefore, mHypoA-2/10-CRE cells represent an ideal model to analyse MC4R-induced CRE activation in a native environment. As shown in Fig. 3E (Suppl. S3), both EPAC-1/2 inhibitors blocked ~80% of α-MSH-induced CRE activation in mHypoA-2/10-CRE cells, in accord with our data obtained in HEK-293 and GT1-7 cells. The selective EPAC-2 inhibitor was without effect. In contrast to HEK-293 and GT1-7 cells, PKA blockers significantly inhibited α-MSH-induced CRE activation in mHypoA-2/10-CRE cells. However, these effects were rather small and never exceeded 20% inhibition. In order to test whether the role of EPACs in MC4R-induced CRE activation is specific for this type of receptor or affects CRE activation in general, we next tested the effects of ESI-09 and HJC-0197 on serum-induced CRE activation. Inhibition of EPAC activity by either blocker did not affect serum-induced CRE activation (Fig. 3F,G), indicating that EPACs are specifically involved in MC4R-dependent signalling pathways. EPACs act as guanine-exchange factors of the monomeric G proteins Rap-1a, -1b or -2. Thus, EPAC-mediated CRE activation implies that Rap proteins might also be involved. Because no reliable Rap inhibitors are available, we used a Rap-1a selective siRNA approach to down-regulate Rap-1a expression in mHypoA-2/10-CRE cells. As shown in Fig. 3H,I, substantial inhibition of Rap-1a protein expression (~50%) was obtained, accompanied by significant inhibition of α-MSH-induced CRE activation (~30%). In contrast, serum-induced CRE activation remained unaffected, further substantiating the concept that blockade of the EPAC/Rap pathway does not blunt CRE-dependent reporter activity in general, but acts specifically if initiated by MC4R signaling.

### EPAC-mediated ERK-1/2 activation is required for α-MSH-induced CREB phosphorylation and CRE activation

**Figure 4:**
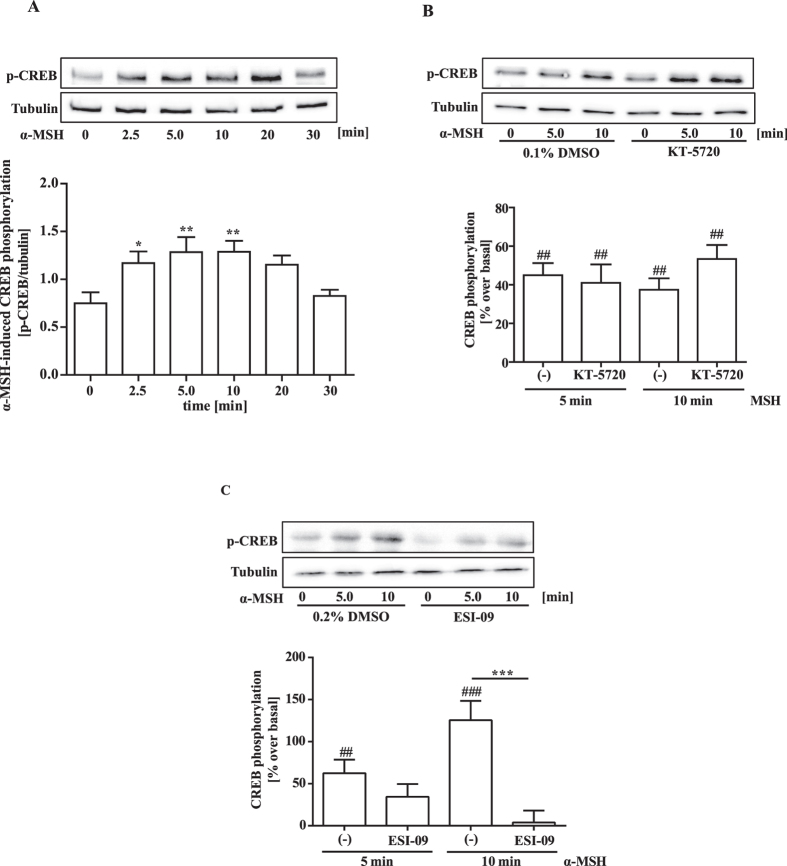
EPAC-1/2 activity is required for α-MSH-induced CREB phosphorylation. In (**A**) Lysates of mHypoA-2/10-CRE cells stimulated with 1 μM of α-MSH for various periods of time were subjected to western-blot analysis using either a phospho-specific antibody against p-CREB (Ser-133) or against the total tubulin protein as a loading control. One representative blot is shown. Data of 12 independent experiments were quantified by densitometry and ratios between p-CREB and tubulin signals calculated. Asteriks indicate a significant difference to time point zero using one-way ANOVA followed by Dunnett’s analysis. In (**B**) lysates of cells preincubated with KT-5720 (5 μM) and in (**C**) with ESI-09 (20 μM) or with the carrier DMSO (0.1% or 0.2% respectively ESI-09) for 30 min and then stimulated with 1 μM α-MSH for 5 and 10 min were subjected to western-blot analysis using either a phospho-specific antibody against p-CREB (Ser-133) or against the total tubulin protein as a loading control. One representative blot is shown. Data of 6 (B) or 8 (**C**) independent experiments were quantified by densitometry, ratios between p-CREB and tubulin signals calculated, α-MSH-induced CREB phosphorylation normalized to not stimulated cells. Hashed signs indicate a significant difference to zero using the one-sample Student’s t-test. Asterisks indicate a significant difference between ESI-09- and DMSO-treated cells using the two-sample Student’s t-test.

**Figure 5:**
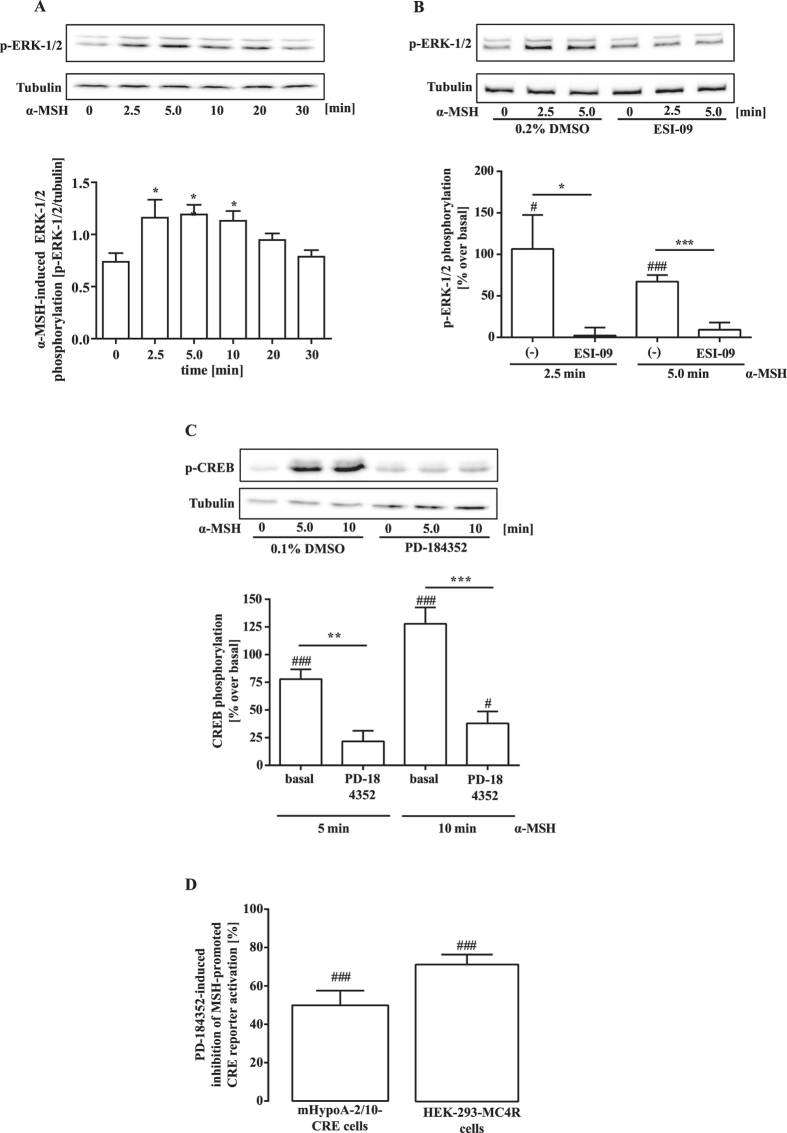
α-MSH-induced CREB/CRE activation requires EPAC-1/2 and ERK-1/2 activity. (**A**) mHypoA-2/10-CRE cells stimulated with 1 μM of α-MSH for various periods of time were subjected to western-blot analysis using either a phospho-specific antibody against p-ERK-1/2 or against the total tubulin protein as a loading control. Data were quantified by densitometry and ratios between p-ERK-1/2 and tubulin signals calculated (N = 13). Asteriks indicate a significant difference using one-way ANOVA followed by Dunnett’s analysis. (**B**) Cells pretreated with ESI-09 (20 μM) or with the carrier DMSO (0.2) for 30 min and then stimulated with 1 μM α-MSH for 2.5 and 5 min were subjected to western-blot analysis using either a phospho-specific antibody against p-ERK-1/2 or against the total tubulin protein as a loading control. Data of 8 (2.5 min) or 10 (5 min) independent experiments were quantified by densitometry, ratios between p-ERK-1/2 and tubulin signals calculated, α-MSH-induced ERK-1/2 phosphorylation normalized to not stimulated cells. Hashed signs indicate a significant difference to zero using the one-sample Student’s t-test. Asterisks indicate a significant difference using the two-sample Student’s t-test. (**C**) Cells pretreated with PD-184352 (10 μM) or with the carrier DMSO (0.1%) for 30 min and then stimulated with 1 μM α-MSH for 5 and 10 min were subjected to western-blot analysis using either a phospho-specific antibody against p-CREB (Ser-133) or against the total tubulin protein as a loading control. Data were quantified by densitometry, ratios between p-CREB and tubulin signals calculated, α-MSH-induced ERK-1/2 phosphorylation normalized to not stimulated cells (N = 6). Hashed signs indicate a significant difference to zero using the one-sample Student’s t-test. Asterisks indicate a significant difference using the two-sample Student’s t-test. In (**D**), mHypoA-2/10-CRE cells or HEK-293-MC4R cells transfected with the CRE reporter plasmid were preincubated with PD-184352 (10 μM) or with the carrier DMSO (0.1%) for 30 min and then stimulated with 1 μM α-MSH for 4 h. Data of 10 (mHypoA-2/10-CRE cells) or 5 (HEK-293-MC4R cells) independent experiments performed in quadruplicates were compiled and shown as inhibition of α-MSH-induced CRE reporter activation in %. Hashed signs indicate a significant difference to zero using the one-sample Student’s t-test.

We next focused on the contribution of EPACs to CREB phosphorylation. Agonist-induced MC4R activation entailed CREB phosphorylation in mHypoA-2/10-CRE cells, reaching a peak about 10 min after stimulation with α-MSH (Fig. 4A). In accord with the CRE activation data, the PKA inhibitor KT-5720 had no inhibitory effect, while the EPAC-1/2 inhibitor ESI-09 fully abrogated α-MSH-induced CREB phosphorylation (Fig. 4B,C). ERK-1/2 kinases induce CREB phosphorylation in non-hypothalamic cells and are activated by EPACs^30^,^31^. Thus, we next investigated α-MSH-induced ERK-1/2 phosphorylation in mHypoA-2/10-CRE cells. Agonist-induced MC4R activation resulted in increased ERK-1/2 phosphorylation for up to 20 min of ligand-stimulation (Fig. 5A). This effect was blocked by ESI-09 (Fig. 5B), implying involvement of EPACs in MC4R-induced ERK-1/2 phosphorylation. These findings support the concept of ERK-1/2 kinases functioning as EPAC-dependent CREB kinases activated by α-MSH. In line with this notion, the ERK-1/2 inhibitor PD-184352 abrogated α-MSH-induced CREB phosphorylation (Fig. 5C), thus highlighting a new signalling pathway linking MC4R-induced cAMP accumulation to CREB phosphorylation by means of EPAC-mediated ERK-1/2 activation. To further corroborate this concept, we tested the effects of PD-184352 on MC4R-induced CRE activation in mHypoA-2/10-CRE and HEK-293-MC4R cells. As shown in Fig. 5D, ERK-1/2 inhibition significantly decreased α-MSH-induced CRE activation in both cell lines, lending further credence to the concept that MC4R-induced CRE activation requires EPAC and ERK-1/2 activity.

### EPACs are necessary for MC4R-promoted c-fos and TRH mRNA induction

**Figure 6:**
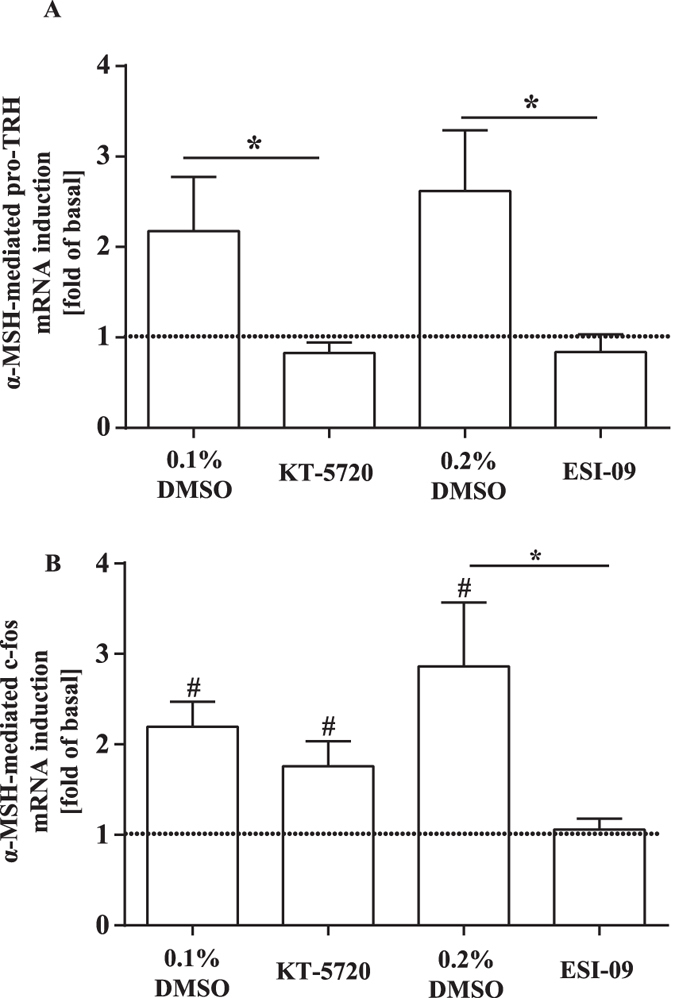
Significant role for EPACs in MC4R-promoted c-fos and TRH mRNA induction. qRT-PCR experiments were performed with cDNAs derived from serum-starved mHypoA-2/10-CRE cells pretreated with KT-5720 (5 μM), ESI-09 (20 μM) or the carrier DMSO (0.1 or 0.2 respectively) for 30 min and then stimulated with 1 μM of α-MSH for 20 min. In (**A**) specific primer pairs against TRH and in (**B**) against c-fos were used. 5 independent cDNAs for each condition were analysed twice in triplicates, data normalized to β-actin and to control cells and expressed as the mean ± S.E.M. Crossing points (Cp) were determined by the software supplied with the LightCycler^®^ 480 and data analysed by the ΔΔCP method (0.5^(target gene–actin)^_MSH_^-(target gene–actin)^_basal_). Asterisks indicate a significant difference between ESI-09 or KT-5720 and DMSO-treated cells using the two-sample Student’s t-test. Hashed signs indicate a significant difference to 1.0 using the one-sample Student’s t-test.

The promoters of the c-fos and TRH gene contain CRE sites and MC4R has been reported to induce both genes^16^,^34^. Thus, we tested the impact of KT-5720 and ESI-09 on MC4R-induced c-fos and TRH mRNA expression in mHypoA-2/10-CRE cells (Fig. 6A,B). The EPAC inhibitor obviated α-MSH-induced expression of both genes, indicating that EPACs play a salient role in MC4R-promoted gene expression. However, the PKA inhibitor only blocked TRH, but not c-fos expression, suggesting that MC4R employ both EPAC- and PKA-dependent signalling pathways to induce TRH, but solely EPAC-dependent pathways to drive c-fos expression.

### EPAC requirement for α-MSH-induced CRE activation is not restricted to the MC4R subtype

**Figure 7:**
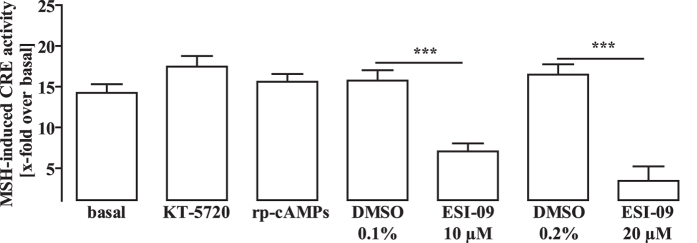
Significant role for EPACs in MC1R-induced CRE activation. B16F10 mouse melanoma cells were transfected with CRE reporter plasmid, serum-starved for 24 h and then stimulated or not with 1 μM of α-MSH for 4 h after 30 min pretreatment with KT-5720 (5 μM), rp-Br-cAMPs (50 μM), ESI-09 (10 or 20 μM) or the carrier DMSO (0.1% or 0.2%). Data of 5 independent experiments performed in quadruplicates were compiled expressed as the mean ± S.E.M. Asterisks indicate a significant difference between ESI-09 or DMSO-treated cells using the two-sample Student’s t-test.

The unexpected central role of EPACs in CRE activation by a prototypical G_s_-coupled receptor such as the MC4R raises the intriguing question as to whether EPACs are also involved in CRE activation engaged by other G_s_-coupled receptors. The MC1R subtype also couples to G_s_ proteins and α-MSH-induced CREB/CRE activation via the MC1R regulates proliferation and differentiation of melanocytes. Very much alike the situation with the MC4R, PKA is thought to play a pivotal role in this process. However, experimental data defining the differential contributions of PKA or EPACs are lacking, even though EPACs have been implicated in melanoma development^35^. To clarify this issue, we employed the B16F10 mouse melanoma cell line endogenously expressing MC1R and investigated the contribution of PKA and EPAC to α-MSH-induced CRE activation (Fig. 7). Of note, EPAC rather than PKA inhibition abrogated CRE activation via MC1R in melanoma cells, supporting the hypothesis that the role of EPAC for melanocortin-induced gene expression is not restricted to the MC4R subtype.

## Discussion

MC4R-induced gene expression via CREs affects multiple body functions such as the central regulation of food intake and energy homeostasis. MC4R-induced CRE activation in the hypothalamus is of prime importance for the regulation of mammalian metabolism, as highlighted by the finding that mutations in the MC4R gene are the most frequent monogenic cause of severe obesity in humans and targeted disruption of the MC4R gene in mice causes an obesity/diabetes syndrome^36^,^37^,^38^,^39^. Although it is commonly accepted that MC4R-induced gene expression mediates biological effects, surprisingly little is known about the signalling pathways underlying MC4R-promoted gene induction. Herein, we uncover a hitherto unappreciated role of EPACs in MC4R-induced gene expression and provide compelling evidence to support the notion that a prototypic G_s_-coupled receptor activates the CREB/CRE pathway via EPACs and not necessarily via PKA.

We dissected the importance of EPACs in MC4R-induced gene expression by taking advantage of the recently introduced EPAC-1/2 inhibitors ESI-09 and HJC-0197 in HEK-293 cells and two hypothalamic cell lines endogenously expressing either human or murine MC4R. Thus, the role of EPACs in MC4R-promoted gene induction does not appear to be restricted to a certain cell-type, but is rather an intrinsic property of the MC4R. A recent study questioned the reliability of ESI-09 and HJC-0197 and described these substances as chemicals with general protein denaturing properties that do not selectively act on EPACs^40^. However, a follow-up study clearly showed that both drugs specifically target EPACs, particularly when used in the low μM range (20 μM for ESI-09 and 25 μM for HJC-0197)^41^. Off-target effects of ESI-09 and HJC-0197 on protein folding in mHypoA-2/10-CRE cells were ruled out, because serum-induced CRE activation (Fig. 3F,G), MC4R-induced cAMP accumulation or bradykinin-induced calcium signaling were left untouched (Suppl. Figs S4 and S5). Hence, both inhibitors appear to be reliable and suitable tools to analyse the biological roles of EPACs in general and to disentangle the role of EPACs in MC4R-induced gene expression in particular^42^,^43^,^44^,^45^,^46^,^47^,^48^,^49^,^50^,^51^.

**Figure 8:**
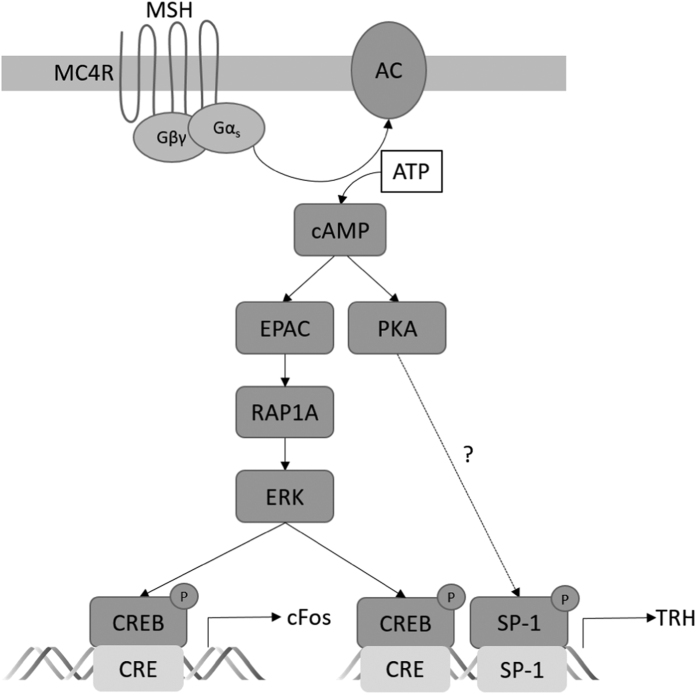
Proposed model of α-MSH-induced signaling leading to c-fos or TRH expression. Signaling compounds required for α-MSH-induced gene expression are depicted. PKA-induced SP-1 phosphorylation is speculative and has not been investigated herein.

Whereas MC4R-promoted CREB phosphorylation has been shown in numerous cell models as well as *in vivo*^20^,^21^,^23^,^26^, the identity of the involved CREB kinase still remained unresolved. In non-hypothalamic cells, ERK-1/2 have been reported to phosphorylate CREB after being activated by EPACs^30^,^31^. Using mHypoA-2/10-CRE cells, we observed that EPAC inhibition blocked MC4R-induced ERK-1/2 and CREB phosphorylation, strongly suggesting ERK-1/2 as the elusive CREB kinases. This conclusion was supported by the finding that inhibition of ERK-1/2 activity mitigated MC4R-promoted CREB phosphorylation and resultant CRE activation. EPACs have been identified as guanine-exchange factors of monomeric Rap GTP-binding proteins. Accordingly, down-regulation of Rap-1a blunted MSH-induced CRE activation. Because Rap proteins have been shown to activate ERK-1/2 in many cell types, our data presented in here advocate the new concept of EPAC/Rap/ERK-1/2 as the molecular link between MC4R and CREB/CRE (Fig. 8).

PKA activity has been proposed to play an important role in MC4R-induced CRE activation. However, experimental data on the effects of PKA inhibition on MC4R-induced CREB phosphorylation or CRE reporter activation are lacking. Herein, we used three structurally and functionally distinct PKA inhibitors and tested their impact on MC4R-induced CRE activation in three different cell lines. We did not observe any inhibitory effects in HEK-293 and GT1-7 cells and only a minor reduction of MC4R-induced CRE activation in mHypoA-2/10 cells. At this point it still remains unclear whether the minor functional consequences of PKA inhibition in mHypoA-2/10-CRE cells are cell type-specific or reflect methodological differences. Of note, our data do not at all exclude a role for PKA in MC4R signalling in general. It has been reported that PKA inhibition by rp-Br-cAMPs or KT-5720 blocks MC4R-mediated expression of the brain-derived neurotrophic factor (BDNF) in rat astrocytes, modulation of the adipose afferent reflex in the hypothalamus and the anorexigenic effects of MC4R in the *nucleus tractus solitarius*^20^,^52^,^53^,^54^. However, it still remains elusive whether or not CREB/CRE is also involved. We noted that KT-5720 significantly decreased TRH, but not c-fos mRNA induction, suggesting that MC4R-dependent PKA activation differentially targets the CRE sites of the TRH, but not of the c-fos promoter. Alternatively, because the TRH promoter also contains binding sites for the transcription factors STAT-3 and SP-1, CREB/CRE-independent signalling might be involved. We have previously shown that MC4R do not activate STAT-3 in mHypoA-2/10 cells calling into question a role for STAT-3 in MC4R-induced TRH expression^24^. However, the SP-1 protein is a substrate for PKA and thus could mediate MC4R-induced TRH expression via PKA independently from CREB^55^,^56^. Interestingly, the promoter of the BDNF gene that is induced by MC4R in a PKA-dependent manner in astrocytes, also contains a site for SP-1 and effects of PKA on the BDNF promoter have been shown to be independent from CREB and CRE, suggesting that MC4R-promoted induction of BDNF via PKA might be mediated by SP-1^57^. However, further studies a required to finally determine the role for PKA in MC4R-induced gene expression and to dissect the underlying signalling cascades.

EPAC proteins have been shown to affect insulin secretion, heart rate, neuronal development, inflammation and cell adhesion^43^. Thus, the aforementioned effects of MC4R signalling on neuroprotection and inflammation overlap with the biological effects of EPACs and may therefore require MC4R-induced EPAC activation *in vivo*. A role of EPACs in the hypothalamic control of food-intake and metabolism has not yet been established, but recent studies showed that EPAC activation leads to leptin resistance and mice lacking the EPAC-1 protein are less sensitive to food-induced adiposity^58^,^59^. Interestingly, it has also been reported that EPAC-1-deficient mice develop increased body weight due to enhanced food intake and a metabolic syndrome similar to mice lacking the MC4R protein^37^,^60^. In spite of various discrepancies, these observations establish the hypothalamic cAMP-EPAC pathway as a central player in the regulation of energy metabolism, either being anabolic via blocking leptin signaling or catabolic via relaying MC4R signals. Because leptin exerts its catabolic effects mainly in the *nucleus arcuatus* and α-MSH in the *PVN*, cell-type specific EPAC signalling may account for these opposing *in vivo* effects. Overall, the present study reveals a hitherto unappreciated role of EPACs for MC4R signalling and hypothalamic gene expression. Therefore, the specific control of hypothalamic EPAC activity may be leveraged as a new strategy for the treatment of severe obesity.

It has been proposed over decades that activation of the cAMP/CREB/CRE pathway by G_s_-coupled receptors regulates central body functions, and in many cases a contribution of PKA has been well documented. However, at least in the case of melanocortin receptors, our data strongly suggest that the role for PKA in CREB/CRE activation has been highly overestimated. It is noteworthy that recently published data even questioned a role for cAMP/CREB/CRE in hypothalamic MC4R signaling at all, because G-protein independent or G_q_-dependent pathways have been shown to be required and mice lacking the CREB protein selectively in the *PVN* exhibited an unaltered reduced food-intake when challenged with a MC4R agonist^61^,^62^,^63^. Thus, further *in vivo* studies are required to reveal the exact physiological role of MC4R-induced CRE activation and TRH induction via EPACs as shown herein.

At this point it is not clear whether or not EPAC-promoted CRE activation is restricted to the MC4R. Of note, melanocortins exert their biological functions by activation of 5 distinct MCR subtypes. Besides the MC4R, the MC1R subtype has attracted much attention because of its significant role in skin pigmentation and melanoma development^64^,^65^. Herein, we also provide first experimental data showing that MC1R-induced CRE activation also requires EPAC and not PKA activity, suggesting that EPACs might play a common role in G_s_-induced CRE activation within the MCR family. In the future it will be enlightening to investigate whether other G_s_-coupled receptors also signal via EPAC and not via PKA to induce CREB/CRE-dependent gene expression.

## Experimental Procedures

### Materials

Cell culture reagents were obtained from Invitrogen (Darmstadt, Germany). The anti-pERK-1/2 (E-4) and the anti-Rap-1a (sc-1482) antiserum was from Santa Cruz (Heidelberg, Germany). The pCREB-Ser-133 antibody was purchased from cell signaling (Leiden, the Netherlands). The anti-tubulin (clone 6-11B-1) and the peroxidase-conjugated anti-mouse or anti-rabbit antibody, both raised in goat, from Bio-Rad (München, Germany). The firefly luciferase substrate was from Promega (Mannheim, Germany). rp-Br-cAMPs and γ-MSH were from SigmaAldrich (Deisenhofen, Germany). ESI-09, ESI-05 and HJC-0197 were from Biolog (Bremen, Germany), A-812511 from abcam (Cambridge, UK) and PD-184,352, HS-024 or NPY from Tocris (Bristol, UK). α-MSH was from Biotrend (Cologne, Germany) and KT-5720 from Enzo life science (Lörrach, Germany).

### Cell culture and transfection

All cells were cultured in DMEM including 10% FBS, 2 mM L-glutamine and penicillin/streptomycin at 37 °C and 5% CO_2_. 18 to 24 h before stimulation, cells were serum-starved in DMEM without FBS. mHypoA-2/10 cells (Clu-176) were purchased from Cedarlane (Burlington, Canada). HEK-293 cells stably expressing the human express-epitope-tagged MC4R receptor and mHypoA-2/10 cells stably expressing the CRE reporter were previously reported^24^,^32^. To down-regulate Rap-1a expression a specific siRNA’s (cat#: AM16708) and one random siRNA construct (cat#: AM4611) were purchased from life technologies. To obtain sufficient transfection efficacy siRNA’s were entered into mHypoA-2/10-CRE cells via electroporation using the Neon^®^ transfection system also from Invitrogen according to the manufacturer’s protocol. Briefly, for one pulse 500,000 cells together with 50 nM of the corresponding siRNA were challenged with 1450 V for 30 ms and then placed on a 6-well cavity for western-blotting or 96-well plates for the reporter assay.

### CRE reporter assay after transient transfection

HEK-293-MC4R, GT1-7 or B16F10 cells were seeded in 24-well plates (~20,000/well) 24 h prior to the experiment and transfected with 250 ng of the luciferase reporter genes plasmid containing six CRE (5′-TGACCTCAC-3′) sites using the Turbofect reagent (#R0531) from ThermoScientific according to the manufacturers’ protocol the next day. After removal of serum for 24 h cells were stimulated for 4 h, lysed (25 mM Tris/HCl pH 7.4, 4 mM EGTA, 8 mM MgCl_2_, 1 mM DTT and 1% Triton-X-100) and luciferase activity measured in white bottom 96-well plates after automatically injecting luciferase substrate. Resulting total light emission was detected every s for 10 s post injection in a FLUOstar^®^ Omega plate reader.

### Reporter assay in mHypoA-2/10-CRE cells

mHypoA-2/10-CRE cells (~15,000 per well) were directly seeded on white bottom 96-well. After 24 h, cells were serum-starved for 24 and stimulated for 4 h. After cell lysis, luciferase activity was measured after automatically injecting luciferase substrate. Resulting total light emission was detected every s for 10 s post injection in a FLUOstar^®^ Omega plate reader.

### cAMP accumulation

To determine agonist-induced cAMP accumulation, ~50,000 cells were seeded in 12-well dishes 24 h prior to the experiment and labelled in serum-free DMEM containing 2 μCi/ml of [^3^H]adenine for 4 h. Cells were stimulated for 30 min at 37 °C in DMEM containing 1 μM IBMX and MSH. The reaction was terminated by removing the medium and adding ice-cold 5% trichloroacetic acid. [^3^H]ATP and [^3^H]cAMP were then purified by sequential chromatography (dowex-resin/aluminium oxide columns), and the accumulation of [^3^H]cAMP was expressed as the ratio of [^3^H]cAMP/([^3^H]cAMP + [^3^H]ATP).

### Western-blot

Cells were seeded on 6-well plates (~100,000/well), cultured for one day, serum-starved for 24 h the next day, stimulated for the indicated period of time with α-MSH and then lysed in Laemmli buffer. Lysates were subjected to SDS-PAGE (10%) and proteins transferred to nitrocellulose by western blotting. For detection of ERK-1/2 or CREB phosphorylation blots were separated by a horizontal cut at ~50 kDa. The lower part was used to analyse ERK-1/2 or CREB phosphorylation with phospho-specific antisera and the upper part to detect total tubulin proteins as a loading control. Immune-reactivity was quantified by densitometry, ratios between p-CREB or p-ERK-1/2 and tubulin signals were calculated, and ligand-induced protein phosphorylation normalized to not stimulated cells.

### Quantitative RT-PCR (qRT-PCR)

Serum-starved mHypoA-2/10-CRE cells were stimulated for 20 min with 1 μM α-MSH in serum-free medium. KT-5720 (5 μM), ESI-09 (20 μM) or DMSO (0.1 or 0.2% respectively) was pre-incubated for 30 min. Stimulation was terminated by rapid cooling on ice and total RNA was isolated using the TriFast^®^ reagent (Invitrogen, Darmstadt, Germany) according to the manufacturer’s instructions. First strand synthesis was carried out with oligo(dT)_18_ primer using 2 μg of total RNA and the RevertAid^™^ H Minus First Strand cDNA Synthesis Kit (Fermentas, Sankt-Leon Roth, Germany). qRT-PCR was done using the LightCycler^®^ 480 SybrGreen I Master Mix (Roche, Mannheim, Germany), intron-spanning primer pairs at a final concentration of 1 μM each and 0,08 μl of the first strand synthesis reaction in a LightCycler^®^ 480 (Roche) using the following conditions: initial denaturation for 15 min at 94 °C, 55 cycles of 94 °C for 10 sec, 55 °C for 10 sec and 72 °C for 10 sec. Crossing points (Cp) were determined by the software supplied with the LightCycler^®^ 480 and data analysed by the ΔΔCP method (0.5^(target gene–actin)^_MSH_^-(target gene–actin)^_basal_). Sequences of primer pairs were 5′-ccaaccgcgagaagatga-3′ and 5′-ccagaggcgtacagggatag-3′ for β-actin, 5′-tgcagagtctccaccttgc-3′ and 5′-ggggataccagttagcacga-3′ for TRH and 5′-gggacagcctttcctact-3′ and 5′-gatctgcgcaaaagtcct-3′ for c-fos.

### Statistics

Data were analysed using GraphPad prism and presented as the mean ± SEM. Differences between two groups of raw data were first compared by two-sample Student’s t-test and among multiple groups with one-way ANOVA followed by Dunnett’s analysis (data not shown). Raw data obtained in experiments with chemical inhibitors were analysed using two-way ANOVA followed by Bonferroni analysis (Suppl. S1–3). After normalization of the raw data, normalized data were shown and analysed using either one- or two-sample Student’s t-test or one-way ANOVA followed by Dunnett’s analysis.

## Additional Information

**How to cite this article**: Glas, E. *et al*. Exchange factors directly activated by cAMP mediate melanocortin 4 receptor-induced gene expression. *Sci. Rep.* **6**, 32776; doi: 10.1038/srep32776 (2016).

## Supplementary Material

Supplementary Information
