# Supplementary material for: Exchange factors directly activated by cAMP mediate melanocortin 4 receptor-induced gene expression
Source: Sci Rep. 2016 Sep 9;6:32776. doi: 10.1038/srep32776 (PMC5017209; doi:10.1038/srep32776)
Supplement: Supplementary Information [file srep32776-s1.pdf]

Supporting Information

Exchange factors directly activated by cAMP mediate melanocortin 4 receptor-induced gene expression

Evi Glas, Harald Mückter, Thomas Gudermann and Andreas Breit*

[1]Walther-Straub-Institut für Pharmakologie und Toxikologie, Ludwig-Maximilians-Universität

*correspondent author: Dr. Andreas Breit, Walther-Straub-Institut für Pharmakologie und Toxikologie, Ludwig-Maximilians-Universität München, 80336 München, Germany, Phone: 0049-89-2180-75755, Fax: 0049-89-2180-75721, e-mail: andreas.breit@lrz.uni-muenchen.de

Table of Contents

Figure S1	α-MSH-induced CRE activation in HEK-293-MC4R cells: raw data
Figure S2	α-MSH-induced CRE activation in GT1-7 cells: raw data
Figure S3	α-MSH-induced CRE activation in mHypoA-2/10-CRE cells: raw data
Figure S4	α-MSH-induced cAMP accumulation in mHypoA-2/10-CRE cells after incubation of the cells with the EPAC inhibitor ESI-09
Figure S5	Bradykinin-induced calcium signaling in mHypoA-2/10-CRE cells after incubation of the cells with the EPAC inhibitor ESI-09

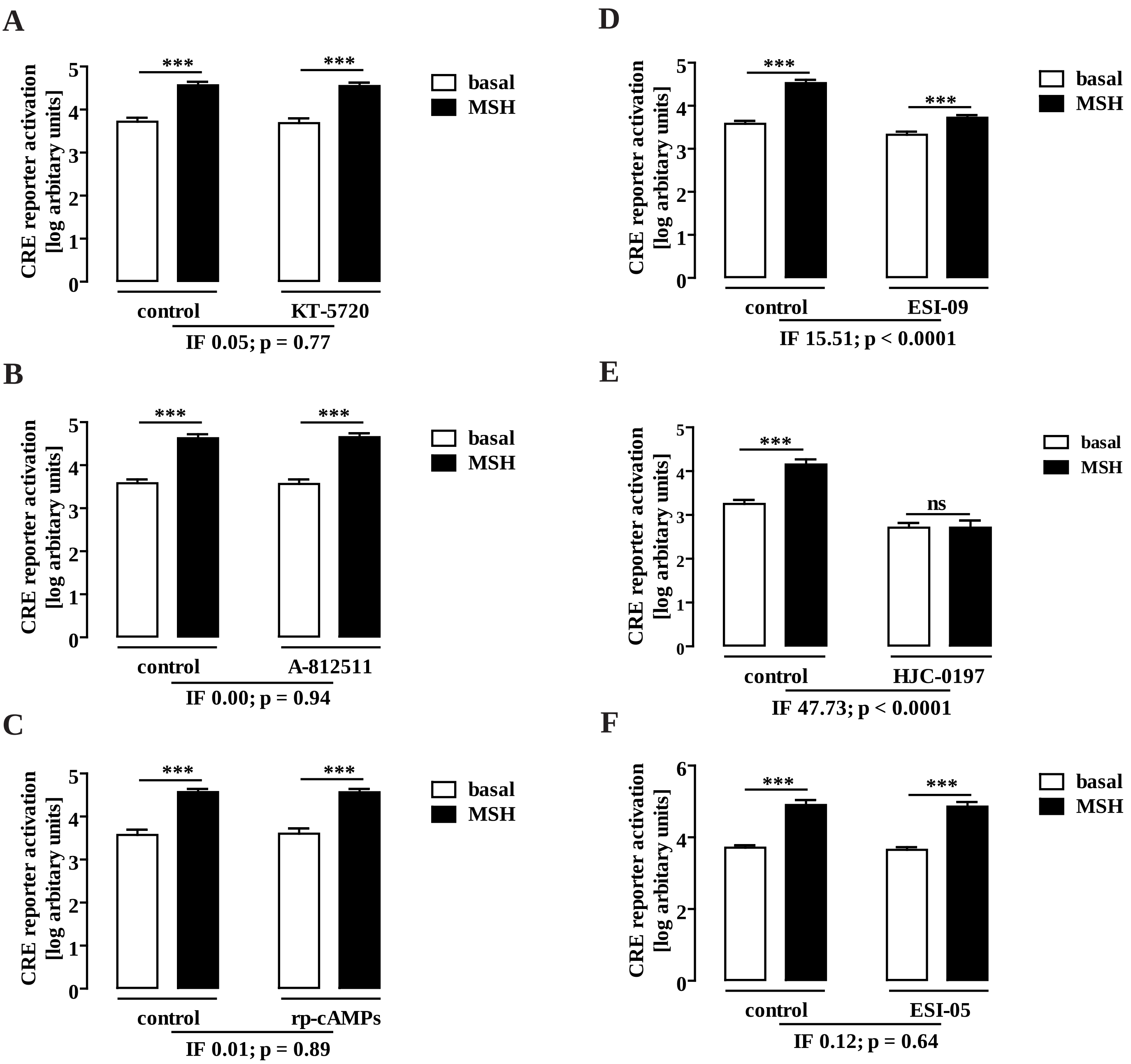

Figure S1: HEK-293-MC4R cells were seeded in 24-well plates (~20,000/well) 24 h prior to the experiment and transfected with 250 ng of the luciferase reporter genes plasmid containing six CRE (5′-TGACCTCAC-3′) sites using the Turbofect reagent (#R0531) from ThermoScientific according to the manufacturers` protocol the next day. After removal of serum for 24 h cells were stimulated for 4 h, lysed (25 mM Tris/HCl pH 7.4, 4 mM EGTA, 8 mM $MgCl_2$, 1 mM DTT and 1 % Triton-X-100) and luciferase activity measured in white bottom 96-well plates after automatically injecting luciferase substrate. Resulting total light emission was detected every s for 10 s post injection in a FLUOstar® Omega plate reader. Normalized data sets shown in Fig 1C are here shown as raw data (log arbitrary light units). Cells were stimulated or not with 1 µM of α-MSH for 4 h after 30 min pretreatment with KT-5720 (5 µM; N=7), A-812511 (10 µM; N=8), rp-Br-cAMPs (50 µM; N=6), ESI-09 (20 µM; N=14), HJC-0197 (25 µM; N=3) or ESI-05 (50 µM; N=4) or the carrier DMSO (0.1 % or 0.2 %). Data were compiled, presented as the mean SEM and analyzed by two-way ANOVA followed by Bonferroni posttests. The interaction factor (IF) obtained by two-way ANOVA analysis describes the interaction between the control and the inhibitor group. Asterisks indicate a significant difference based on the Bonferroni posttest between α-MSH stimulated cells and basal.

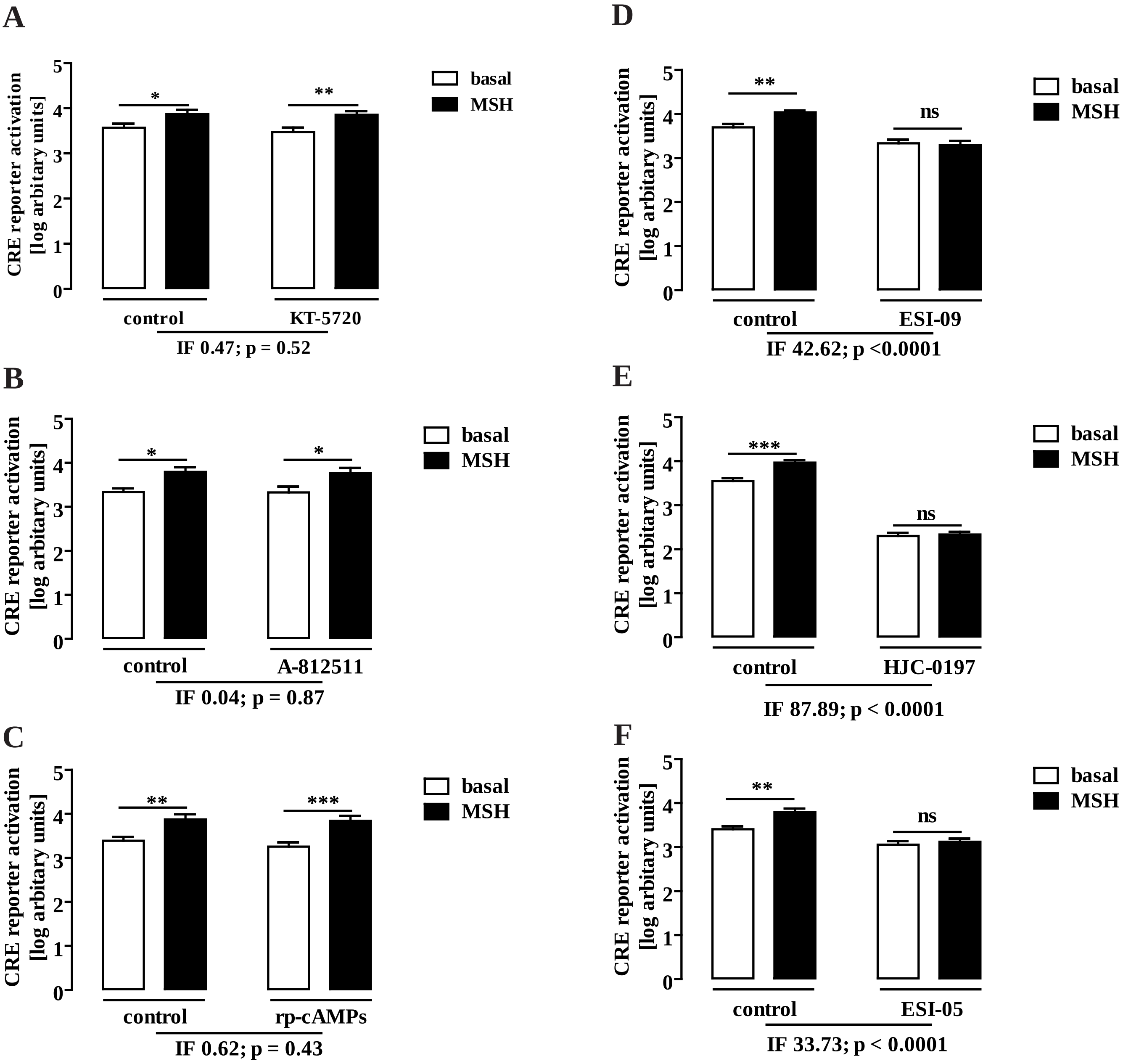

Figure S2: GT1-7 cells were seeded in 24-well plates (~20,000/well) 24 h prior to the experiment and transfected with 250 ng of the luciferase reporter genes plasmid containing six CRE (5´-TGACCTCAC-3´) sites using the Turbofect reagent (#R0531) from ThermoScientific according to the manufacturers` protocol the next day. After removal of serum for 24 h cells were stimulated for 4 h, lysed (25 mM Tris/HCl pH 7.4, 4 mM EGTA, 8 mM $MgCl_2$, 1 mM DTT and 1 % Triton-X-100) and luciferase activity measured in white bottom 96-well plates after automatically injecting luciferase substrate. Resulting total light emission was detected every s for 10 s post injection in a FLUOstar® Omega plate reader. Normalized data sets shown in Fig 2B are here shown as raw data (log arbitrary light units). Cells were stimulated or not with 1 µM of α-MSH for 4 h after 30 min pretreatment with KT-5720 (5 µM; N=7), A-812511 (10 µM; N=4), rp-Br-cAMPs (50 µM; N=5), ESI-09 (20 µM; N=5), HJC-0197 (25 µM; N=3) or ESI-05 (50 µM; N=5) or the carrier DMSO (0.1 % or 0.2 %). Data were compiled, presented as the mean SEM and analyzed by two-way ANOVA followed by Bonferroni posttests. The interaction factor (IF) obtained by two-way ANOVA analysis describes the interaction between the control and the inhibitor group. Asterisks indicate a significant difference based on the Bonferroni posttest between α-MSH stimulated cells and basal.

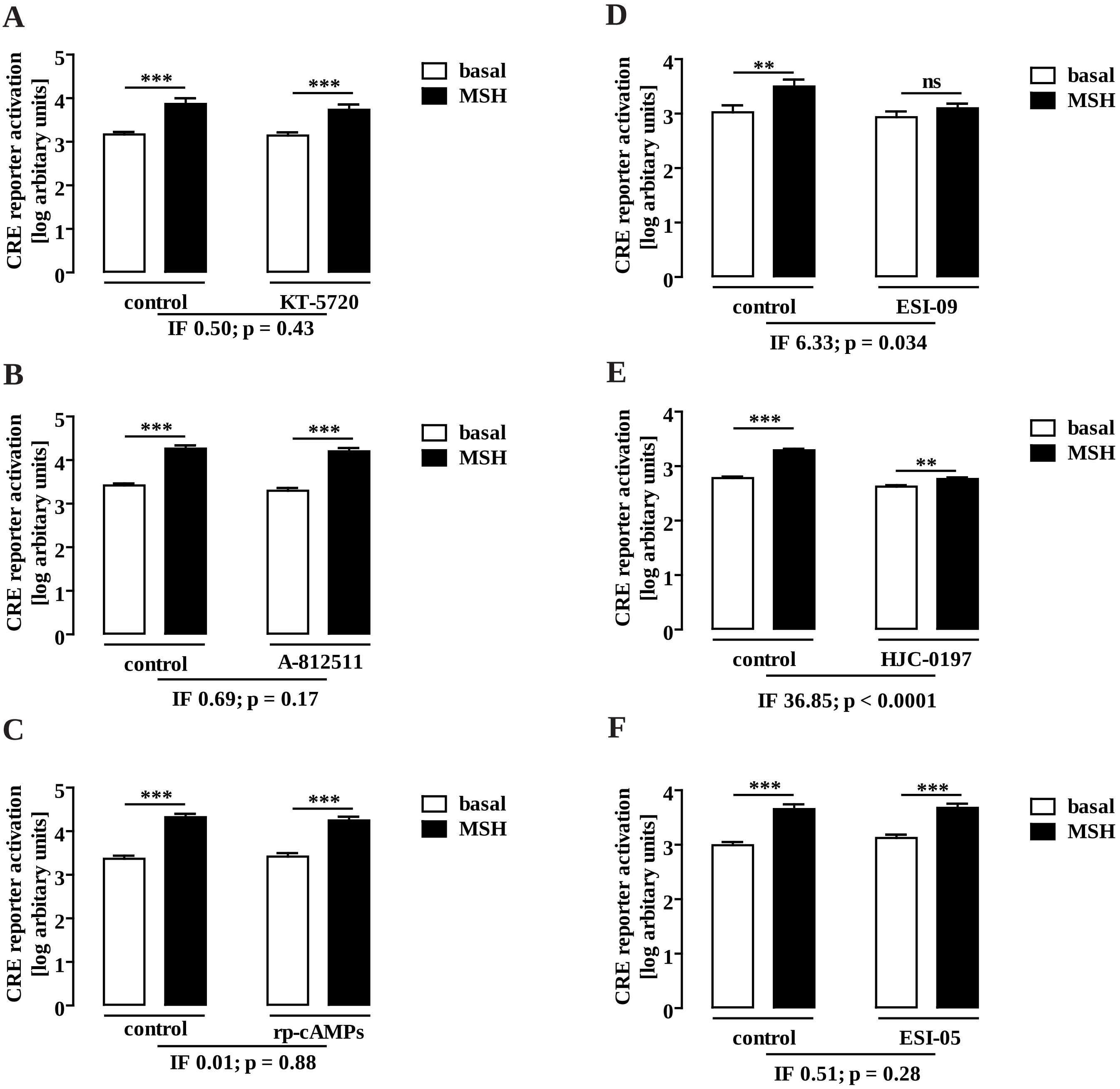

Figure S3: mHypoA-2/10-CRE were seeded in 24-well plates (~20,000/well) 24 h prior to the experiment. After removal of serum for 24 h cells were stimulated for 4 h, lysed (25 mM Tris/HCl pH 7.4, 4 mM EGTA, 8 mM $MgCl_2$, 1 mM DTT and 1 % Triton-X-100) and luciferase activity measured in white bottom 96-well plates after automatically injecting luciferase substrate. Resulting total light emission was detected every s for 10 s post injection in a FLUOstar® Omega plate reader. Normalized data sets shown in Fig 2B are here shown as raw data (log arbitrary light units). Cells were stimulated or not with 1 µM of α-MSH for 4 h after 30 min pretreatment with KT-5720 (5 µM; N=6), A-812511 (10 µM; N=11), rp-Br-cAMPs (50 µM; N=5), ESI-09 (20 µM; N=14), HJC-0197 (25 µM; N=5) or ESI-05 (50 µM; N=10) or the carrier DMSO (0.1 % or 0.2 %). Data were compiled, presented as the mean SEM and analyzed by two-way ANOVA followed by Bonferroni posttests. The interaction factor (IF) obtained by two-way ANOVA analysis describes the interaction between the control and the inhibitor group. Asterisks indicate a significant difference based on the Bonferroni posttest between α-MSH stimulated cells and basal.

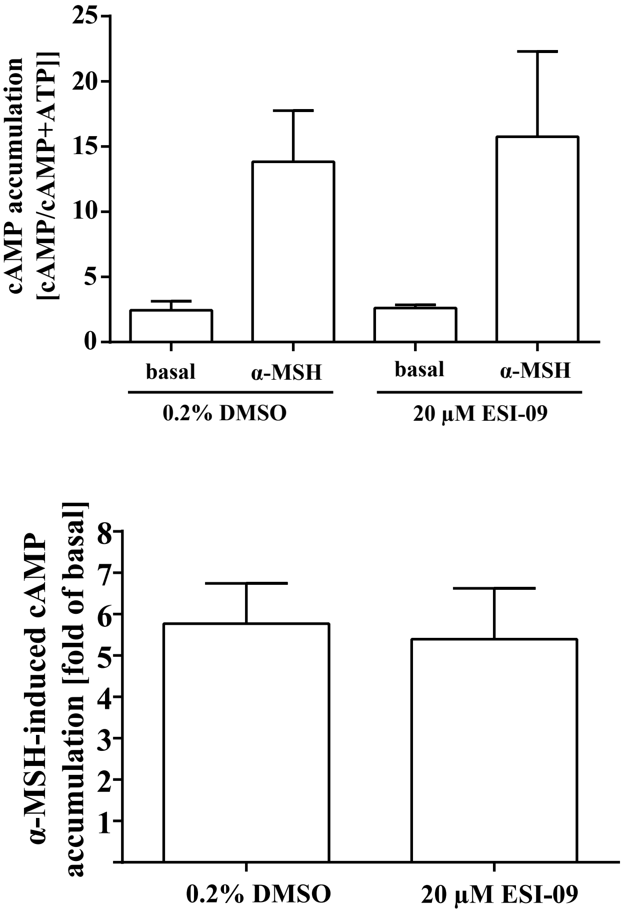

Figure S4. α-MSH-induced cAMP accumulation was measured in mHypoA-2/10-CRE cells after incubation of the cells with the EPAC inhibitor ESI-09 or the carrier control DMSO. To determine agonist-induced cAMP accumulation, ~50,000 cells were seeded in 12-well dishes 24 h prior to the experiment and labelled in serum-free DMEM containing 2 µCi/ml of [3H]adenine for 4 h. Cells were stimulated for 30 min in DMEM containing 1 µM IBMX and MSH. The reaction was terminated by removing the medium and adding ice-cold 5 % trichloroacetic acid. [3H]ATP and [3H]cAMP were then purified by sequential chromatography (dowex-resin/aluminium oxide columns), and the accumulation of [3H]cAMP was expressed as the ratio of [3H]cAMP/([3H]cAMP + [3H]ATP).

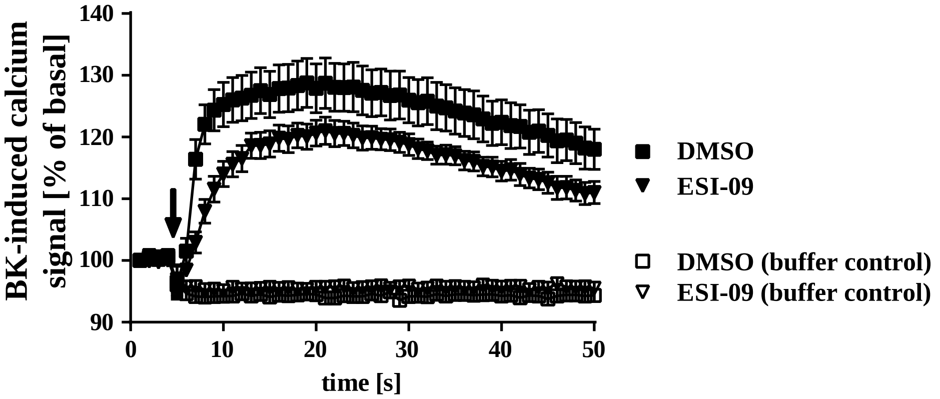

Figure S5. Bradykinin (BK)-induced calcium signaling was measured in mHypoA-2/10-CRE cells after incubation of the cells with the EPAC inhibitor ESI-09 or the carrier control DMSO. 24 h prior cells were seeded in a 10 cm dish and then loaded with 10 µM fura-2-AM in HBS buffer for 30 min at 37 ºC. Labelling of cells was improved by adding 0.02 % pluronic F-127 to the labelling buffer. After harvesting the cells in HBS, ~100,000 cells per well were seeded in 96-well plates and fluorescence measured in a FLUOstar® Omega plate reader from BMG (Offenburg, Germany) at 37 ºC. HBS as a control or HBS including the corresponding ligand were automatically injected 5-10 s after starting the measurement. In intervals of 1.14 s total emission (520 ± 20 nm) was measured after excitation of the sample with 340 ± 15 nm or 380 ± 15 nm. Fura-2-ratios (340/380) were then plotted against the time in seconds, after setting the first ratio measured to 100 %.
